# A case report: hemothorax caused by rupture of the left atrial appendage

**DOI:** 10.1186/s40792-016-0270-2

**Published:** 2016-11-26

**Authors:** Hiroaki Oizumi, Kenji Suzuki, Hironobu Hoshino, Takahiro Tatsumori, Hideomi Ichinokawa

**Affiliations:** 1 Department of General Thoracic Surgery, Juntendo University Shizuoka Hospital, 1129, Nagaoka, Izunokuni, Shizuoka 410-2295 Japan; 2 Department of General Thoracic Surgery, Juntendo University School of Medicine, Tokyo, Japan

**Keywords:** Hemothorax, Blunt cardiac rupture, Pericardial effusion, Chest surgery

## Abstract

Cardiac rupture is defined as a full-thickness myocardial tear; this injury after blunt chest trauma is rare, and is associated with high mortality. Blunt cardiac rupture typically presents with either cardiac tamponade or massive hemothorax, and is often unrecognized in the context of blunt chest trauma. It is a little known fact that pericardial effusions can decrease due to pericardial lacerations. Hence, cardiac rupture with pericardial lacerations may be easily overlooked especially by chest surgeons. We herein report a case of hemothorax caused by rupture of the left atrial appendage. An 80-year-old male was involved in a motor vehicle crash. We made the diagnosis of hemothorax on the basis of bloody thoracic effusion and left pleural effusion on computed tomography (CT). CT also showed small pericardial effusion in amount and non-displaced rib fractures. We made a tentative diagnosis of intercostal artery injury with rib fractures, we performed left thoracotomy. However, in the operating room, we recognized that cardiac rupture led to massive hemothorax, and that hemothorax was not associated with intercostal artery injury. We repaired left atrial appendage rupture, and his postoperative course was uneventful. Cardiac rupture can present as slight pericardial effusion with hemothorax. On the basis of this case, we propose that cardiac rupture should be considered at the time of hemothorax examination with careful attention to pericardial effusions.

## Background

Cardiac rupture is defined as a full-thickness tear of the cardiac wall, involving all the layers [1]. Blunt cardiac rupture is a rare entity, the frequency of blunt cardiac rupture among hospital trauma admissions ranges from nearly 0.16 to 2% [2]. Approximately 10% of patients who suffer blunt injury to the chest have cardiac rupture [3]. An overall mortality of cardiac rupture patients is equal to 93%, and this high incidence of mortality is related to overlooked injuries [1, 4]. Blunt cardiac rupture characteristically presents with either pericardial tamponade or massive hemothorax [5]. Cardiac rupture patient who have not pericardial tears produce cardiac tamponade. Cardiac rupture patient who have pericardial tears presents as hemothorax, because pericardial tears avoid gathering of intra-pericardial fluid [2, 6]. When the chest surgeons estimate the origin of bleeding, it is difficult for them to suspect cardiac rupture, and delayed diagnosis leads to poor prognosis. We present a case of blunt cardiac rupture with pericardial lacerations causing hemothorax. Our preoperative assessment of bleeding point was intercostal arteries. However, during our surgery, we recognized that massive hemothorax was related not to rib fractures but to cardiac rupture. We treated the present case as a warning signal for hemothorax.

## Case presentation

**Fig. 1:**
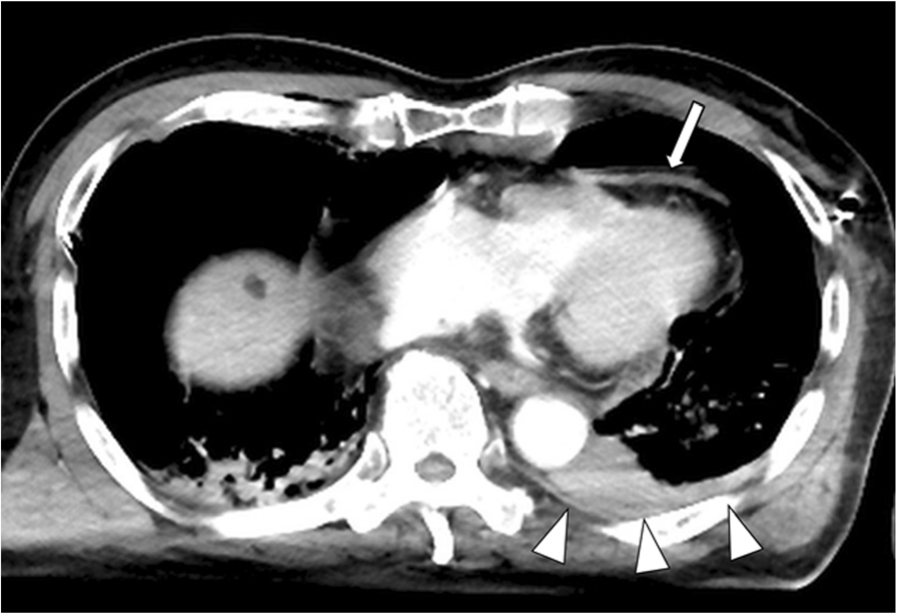
Initial chest computed tomography. Computed tomogram showed slight pericardial effusion (*arrow*). Computed tomogram showed left hemothorax with extravasation of contrast (*arrow heads*)

An 80-year-old male was involved in a two-car collision as a driver of one of the vehicles. His seat belt was fastened. Physical examination revealed a blood pressure of 70/30 mm Hg, a heart rate of 83 bpm, oxygen saturation 70%, and decreased vesicular sounds. He was intubated, and he was treated with bilateral thoracotomy tube placement. Bloody thoracic effusion was confirmed by a left thoracocentesis. Approximately 450 ml of sanguineous effusion was drained by left thoracocentesis. He was immediately transported to Juntendo University Shizuoka Hospital. Thorough transportation, 200 ml of bloody thoracic effusion was drained subsequently. On arrival, hemoglobin was 10.2 g/dl. Chest radiograph revealed a normal mediastinum. His Focused Assessment with Sonography for Trauma (FAST) window was negative for fluid except left pleural effusion. Total body CT scan demonstrated left hemothorax with extravasation of contrast and slight pericardial effusion without extravasation of contrast (Fig. 1). Left rib fractures, slight lung contusion in the left lower lobe, and clinically not significant left pneumothorax were also seen by the CT. Fifth and sixth rib fractures were not displaced. The patient was transferred to the intensive care unit, we used non-operative management in watchful waiting, because hemorrhage volume did not reach the criterion for operability and hemodynamic states were stable at first.

**Fig. 2:**
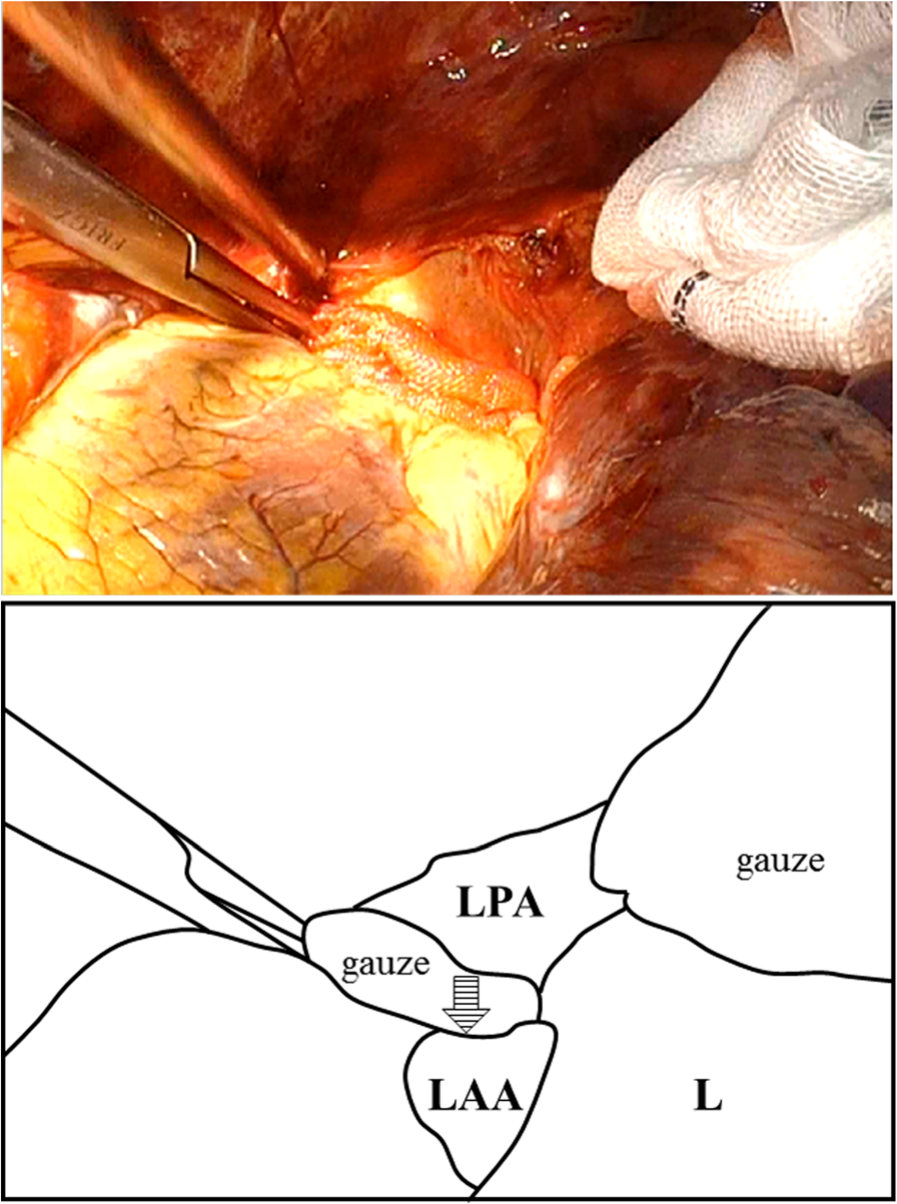
Intraoperative photograph and its schematic illustration. Left appendage bleeding could be controlled by astriction. Bleeding point (*arrow*). LAA, left atrial appendage; LPA, left pulmonary artery; L, Lung

We took care of the patient in ICU for 3 h. We gave transfusion of red blood cells (12U) in order to maintain blood pressure of 100/60 mm Hg and a heart rate of 70 bpm and to maintain hemoglobin level of at least 10.0 g/dl for 3 h. This clinical condition meant hemodynamically unstable, we decided to perform thoracic surgical exploration in the operating room. We predicted that the sources of hemorrhage were the fifth and sixth intercostal arteries and lung contusion in the left lower lobe adjacent rib fractures. We performed left posterolateral thoracotomy, so that we could extend this incision. Thoracotomy revealed bloody effusion and clots, but revealed absence of intercostal artery injury. We identified large amount of fresh blood and clots below the left proximal pulmonary artery, and we identified pericardial lacerations which ran about 30 mm. Then, we extended the incision to anterolateral by about 10 cm. We observed this area carefully to confirm that fresh blood emerged from pericardial space. Then, we dissected pericardium of 50 mm in diameter in order to detect the source of hemorrhage. We detected that a single left appendage laceration ran about 10 mm. Left appendage bleeding could be controlled by astriction (Fig. 2). We evacuated approximately 1500 ml of fresh blood and clots from the left thoracic cavity. Subsequently, we compressed bleeding point with Tissue Sealing sheet (TachoSil®, Tokyo, Japan) for 15 min. Exploration of heart and left pleural cavity did not show other injuries such as circumflex coronary artery injury and lung contusion and diaphragm injury. The patient’s postoperative course was uncomplicated. Two pleural drains were removed on postoperative day (POD) 7 and on POD 15, respectively. The patient was discharged on POD 49 without a sign of pleural effusion.

### Discussion

Cardiac rupture is an injury to the wall of the heart, usually associated with blunt trauma. The condition is rare, occurring in less than 2% of trauma patients in most reports, and the mortality remains high, which is generally attributed to failure to diagnose the condition. Quick diagnosis and repair of cardiac rupture are significant to the optimization of outcome [7].

Cardiac rupture is classified into two categories depending on pericardial laceration. Thirty percent of cardiac ruptures are accompanied with pericardial lacerations [8], which leads to hemothorax. Seventy percent of the cardiac ruptures are accompanied with cardiac tamponade. Usually, chest surgeons perform examinations of hemothorax at emergency department. Thus, chest surgeons must recognize the possibility of cardiac rupture with pericardial lacerations, which can produce a clinical picture of hemothorax.

This patient course provided one important clinical suggestion. Pericardial effusion is useful for building clinical reasoning. We can suspect cardiac rupture as the cause of hemothorax based on pericardial effusion, even if those are small in amount. Cardiac rupture patients who have pericardial tears present as hemothorax, because pericardial lacerations avoid gathering of intra-pericardial fluid [2, 6]. To the best of our knowledge, 11 previous alive cases of blunt cardiac rupture with concurrent hemothorax have been reported (Table 1). Pericardial effusion on computed tomography (CT) was detected in six cases (54%). The other reports (46%) did not mention existence or non-existence of pericardial effusion. Pericardial effusion may be useful to predict cardiac rupture, even if there are small amount of pericardial effusion. In our case, pericardial effusions are little and there is no extravasation of contrast in the pericardial sac. Contrast collection in the pericardial space has unknown specificity of cardiac rupture in previous reports. Therefore, we must focus on pericardial effusions for rapid diagnosis and treatment when examining a hemothorax patient.

**Table 1:** Alive cases of hemothorax caused by cardiac rupture

References	Age/sex	Triggering event	Pericardial effusion (on CT)	Cardiac lesion	Surgical incision	Operation	Outcome
Sliker [11]	59/F	Motor vehicle crash	+	Left ventricle	Left thoracotomy	NR	Alive
Yamaguchi [12]	59/M	Motor vehicle crash	NR	Left atrial appendage	Median sternotomy	Suture	Alive
Ball [1]	65/F	Motor vehicle crash	+	Right ventricle	Median sternotomy	Suture	Alive
Agathe [4]	72/M	Motor vehicle crash	+	Right ventricle	Median sternotomy	Suture	Alive
Agathe [4]	45/M	Motor vehicle crash	+	Right atrial appendage	Right posterolateral thoracotomy	Suture	Alive
Kawahira [13]	31/F	Fell down the stairs	+	Right atrial appendage, right ventricle	Median sternotomy	Suture	Alive
Murakami [14]	24/F	Motor vehicle crash	NR	Right atrial appendage	NR	Suture	Alive
Kuroda [15]	71/F	Fell down the stairs	+	Right atrium	Median sternotomy	Suture	Alive
Miki [16]	41/M	Motorcycle crash	NR	Right atrial appendage	Clamshell	Suture	Alive
Miki [16]	75/F	Motorcycle crash	NR	Left atrial appendage	Clamshell	Suture	Alive
Baker [6]	46/F	Motor vehicle crash	NR	Left atrium, left ventricle	Left thoracotomy	Suture	Alive
Our study	80/M	Motor vehicle crash	+	Left atrial appendage	Left posterolateral thoracotomy	Astriction (suture less)	Alive

*NR* not reported

We should immediately examine sources of bleeding through thoracotomy if bleeding points are unclear on CT. Thoracotomy prove to be both diagnostic and theoretically therapeutic [7]. When the patient has massive bleeding unexplained by the estimated bleeding point, thoracotomy must be done. A question arises as to the option of incision. Once cardiac rupture is strongly suspected, incision is basically rapid, especially in cardiac arrest. Left anterolateral thoracotomy is speedy but it gives very poor access to any cardiac structure except the apex of the left ventricle [9]. In addition, the extension of this incision across the sternum into a clamshell (bilateral anterior thoracotomies) leads to four more bleeding vessels (internal mammary arteries and veins) to contend with [9]. However, in our case, we ought to have chosen the left anterolateral thoracotomy in supine position for an exploration of the left thorax, because the advantage of the left anterolateral thoracotomy is that the incision might be extended to the right if we needed to explore the right thorax.

A query arises about the option of place at which we operate thoracotomy. For the rapidly decompensating or arresting patient, emergency thoracotomy, i.e., left anterolateral thoracotomy in an emergency room has proven to be life-saving [7]. Even if the patient is hemodynamically stable, initial drainage of more than 1000 mL or constant bleeding is at a rate more than 200 ml/h for four consecutive hours from an inserted thoracic drain [10], then we must make thoracotomy prior to cardiac arrest. Meanwhile, “200 ml/h” is one of the indicators, because in our case drainage function from the chest tube was interfered by clot formation. Moreover, our patient might have a chance to go to the operating room earlier in order to stop the bleeding.

## Conclusions

In summary, we reported a case of hemothorax secondary to blunt cardiac rupture. Pericardial effusion is crucial for suspecting cardiac rupture as a cause of hemothorax, even if it is slight, because cardiac rupture without prompt recognition and proper treatment results in high mortality.

## Abbreviations

CT: Computed tomography
FAST: Focused Assessment with Sonography for Trauma
ICU: Intensive care unit
POD: Postoperative day
